# Method for identification of 10 SSR markers from monkey genomes and its statistical inference with One & Two-way ANOVA

**DOI:** 10.1016/j.mex.2022.101833

**Published:** 2022-08-30

**Authors:** Chinta Someswara Rao, G.N.V.G. Sirisha, K. Butchi Raju, N V Ganapathi Raju

**Affiliations:** a Department of CSE, SRKR Engineering College, Bhimavaram, AP, India; b Department of CSE, GRIET, TS, India; c Department of IT, GRIET, TS, India

**Keywords:** SSRs, Monkey, Pattern matching, Genomes, SSR, Simple sequence repeats, ANOVA, Analysis of Variance

## Abstract

DNA tracts that include simple sequence repeats (SSRs), sometimes known as genetic "stutters), are composed of a few to many tandem repetitions of a short base-pair motif. These sequences frequently mutate, changing the amount of repetitions. SSRs are frequently found in promoters, untranslated regions, and even coding sequences, therefore these alterations can significantly affect practically every aspect of gene activity. SSR alleles can also contribute to normal diversity in brain and behavioural features. Mutational expansion of certain triplet repeats is the cause of a number of inherited neurodegenerative diseases. Due to its importance in genetic research, in this paper we explored Ten SSR markers TAGA, TCAT, GAAT, AGAT, AGAA, GATA, TATC, CTTT, TCTG and TCTA that are identified from the genomes of Eleven distinct monkeys: A.Nancymaae, C.C.Imitator, C.Atys, M.Leucophaeus, P.Paniscus, R.Bieti, R.Roxellana, S.Boliviensis, T.Syrichta, C.A.Palliatus and M.Nemestrina using pattern matching mechanism. We identified 4bp SSR from eleven monkey dataset's Unchr chromosome mainly in this paper. The proposed approach finds the exact place/location of the SSR's and number of times that it appears in the given genome sequence. The identified patterns are analyzed with One-way and Two-way ANOVA that gives better analysis which is useful for genomic studies. Also, this 4bp Ten SSR markers data is a valuable to illustrate genetic variation of genomic study.

• The great specificity of data sets produced from monkey genomes with pattern matching has been demonstrated.
• These findings show that SSR identification could be a useful tool for determining genome similarity and comparability.
• Researchers can use the raw sequencing data to conduct additional bioinformatics analysis.

## Graphical abstract

**Figure:**
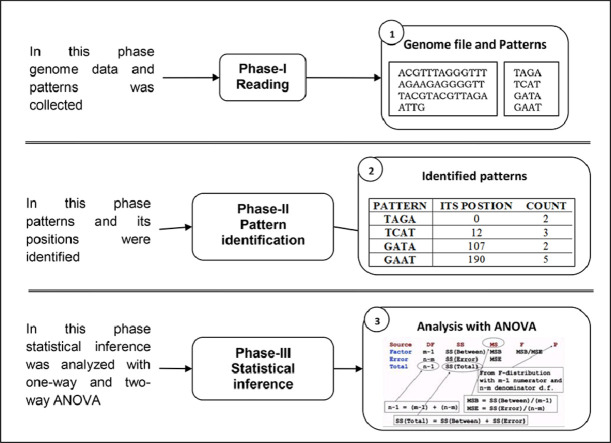

Specifications Table

**Table:** 

Subject area:	Bio-informatics
More specific subject area:	Genomes of monkeys
Name of your method:	SSR identification
Name and reference of original method:	NA
Resource availability:	**Repository name:** Two way ANOVA statistic and *p* value **Data identification number:** 10.17632/w42hmpwvby.2 10.17632/t3msbvj89t.2 **Direct URL to data:** https://data.mendeley.com/datasets/w42hmpwvby/2 https://data.mendeley.com/datasets/t3msbvj89t/2

## Method details

Tandems of repeating DNA sequences are present in various quantities for the majority of genomes in simple sequence repeats (SSRs). This repetition of genetic mapping and population research has been widely employed. SSRs also give molecular tools for the understanding of spatial links between segments of chromosomes which, in turn, help in the analysis of temporal linkages between species and genera.

It is predicted that the study of repeat frequency and their distribution pattern in the genome would assist to comprehend their meaning. There are accumulated indications suggesting SSRs influence gene expression [1], [2], [3].

Complete genome sequences were available for several species and genome-wide analysis were carried out. In this study, we analysed Unchr chromosome of Eleven different monkeys A.Nancymaae, C.C.Imitator, C.Atys, M.Leucophaeus, P. Paniscus, R.Bieti, R.Roxellana, S.Boliviensis, T.Syrichta, C.A.Palliatus and M.Nemestrina and Ten SSR loci were investigated for their spread and frequency of occurrence.

Previously, few studies have tried to evaluate tandem replacement distributions in monkey genomes [4], but they are restricted to a single or a small number of genomes. This multiple mining employing Analysis of Variance (ANOVA) helps to understand and resolve biological issues.

**Fig. 1:**
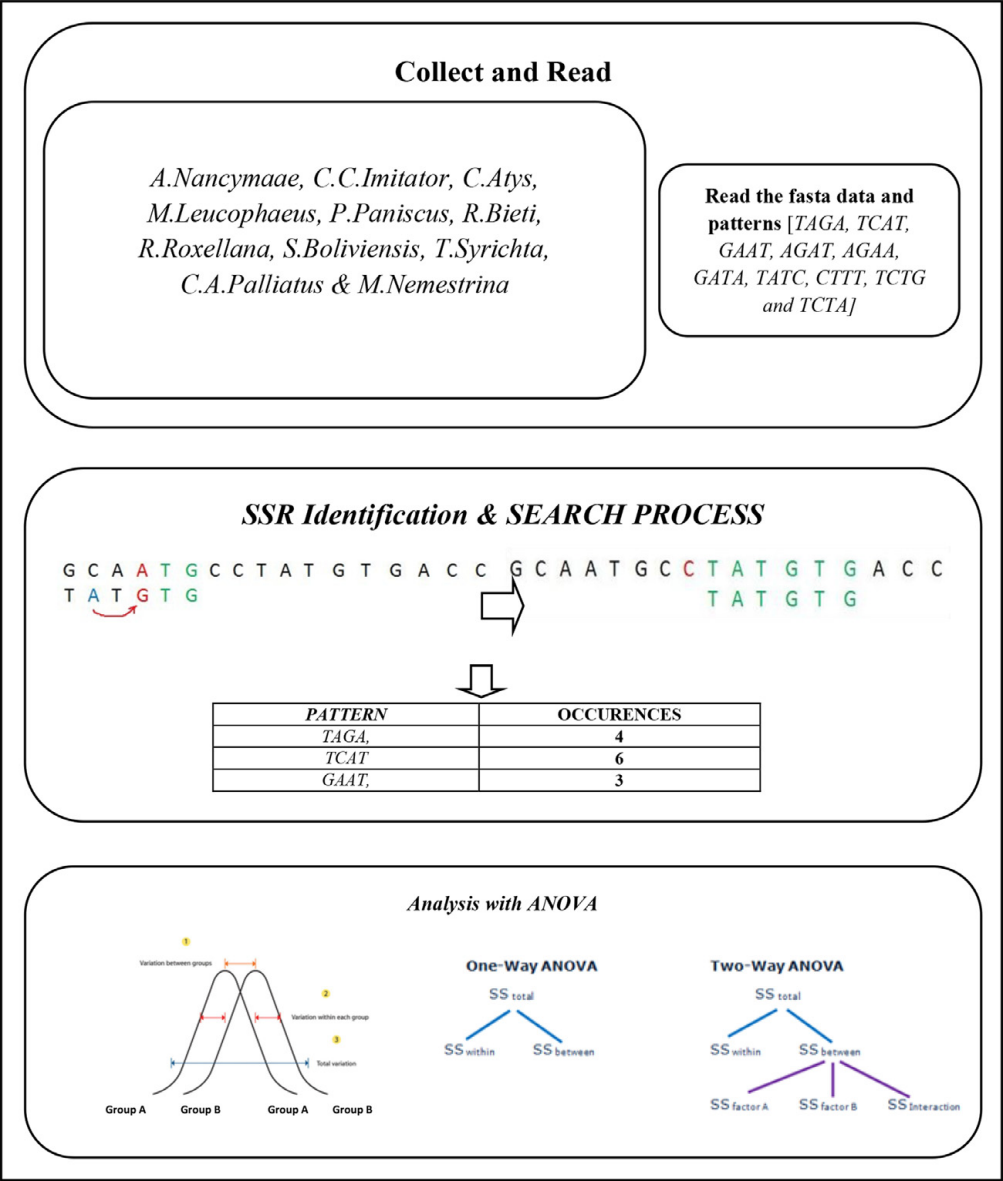
Structure of the model.

The proposed structure of the method is shown in Fig. 1 that comprises of collected data set and read, SSR identification and Search process and Analysis of variance (ANOVA).

Fasta format of A.Nancymaae, C.C.Imitator, C.Atys, M.Leucophaeus, P. Paniscus, R.Bieti, R.Roxellana, S.Boliviensis, T.Syrichta, C.A.Palliatusand M.Nemestrina datasets are collected and Ten patterns are considered for reading.

### SSR Identification

In this paper Unchr chromosome of A.Nancymaae, C.C.Imitator, C.Atys, M.Leucophaeus, P. Paniscus, R.Bieti, R.Roxellana, S.Boliviensis, T.Syrichta, C.A.Palliatus and M.Nemestrina and the ten(TAGA, AGAA, GATA, TCTA, TCAT, GAAT, AGAT, CTTT, TATC, TCTG) SSRs are considered. Using a string matching method, SSRs are retrieved from monkeys. String matching is a search method that looks for repeats in a certain chromosome file.

### Search process

Algorithm 1 describes the complete SSR identification heuristic procedure. In this heuristic procedure, the chromosomes and SSRs are given as input, which then invokes the *first_occurance_position_heuristic, bad_character_heuristic, and good_suffix_heuristic* procedures. Finally, this algorithm displays the pattern and its position and continues the search process until the end of the given chromosome sequence.

Algorithm 2 describes the first occurrence position heuristic procedure. In this heuristic procedure, the pattern's rightmost character, pattern [m-1], was compared with the corresponding character in the genome sequence; if a match is found, the match position is returned; otherwise, the comparison continues until the end of the genome sequence.

Algorithm 3 describes the Bad Character Heuristic Procedure. When the mismatch case occurred, then this heuristic procedure was invoked and returned the shifted position of the pattern. If any of the pattern characters was not matched with the genome sequence, the entire pattern position was shifted; otherwise, the number of characters matched was used to shift the pattern.

Algorithm 4 describes the Good Suffix Heuristic Procedure. This heuristic procedure was invoked at the time of a complete pattern match and returned the search position. Look If a substring of a pattern is matched until a bad character has a good suffix, after a mismatch that causes a negative shift in bad character heuristics, we take a step forward equal to the length of the suffix found.

This procedure is repeated for all SSRs as well as the whole data in the chromosomes.

## Analysis of variance (ANOVA)

The analysis of variance (ANOVA) [5], [6], [7] is a set of statistical models and estimate processes for analyzing differences between group means in a sample. It is useful for comparing (testing) the statistical significance of three or more group means. For this, here we are calculated the $SS_{between},\;MS_{between},\;df_{between},\;SS_{within},\;MS_{within},\;df_{within},\;and\;F_{value}$values. We'll sum them up by multiplying each squared variation by each sample size. For between-group variability, this is known as the sum-of-squares, as shown in Eq. (1).

(1) $$SS_{between}=\;\sum_{i=1}^{k}\begin{array}{cc} n_{i} & {\left(\bar{x_{i}}-\bar{x_{G}}\right)}^{2} \end{array}$$

$MS_{between}$ is calculated with Eq. (2) and $df_{between}$ is calculated with Eq. (3).

(2) $$MS_{between}=\;\sum_{i=1}^{k}\begin{array}{cc} n_{i} & \frac{{\left(\bar{x_{i}}-\bar{x_{G}}\right)}^{2}}{k-1} \end{array}$$

(3) $$df_{beteen}=k-1$$

Within-group variability is measured by how far each sample's value deviates from the sample mean.

$SS_{within}$ is calculated with Eq. (4), $df_{within}$ is calculated with Eq. (5) and $MS_{within}$ is calucalted with Eq. (6)

(4) $$SS_{within}=\sum {\left(x_{ij}-\bar{x_{j}}\right)}^{2}$$

(5) $$df_{within}=N-k$$

(6) $$MS_{within}=\sum \frac{{\left(x_{ij}-\bar{x_{j}}\right)}^{2}}{N-k}$$

### F-Statistic

It assesses the means of two or more samples significance. Their value is less then sample means are close to each other. We can not rule out the null hypothesis in such instance. It is calculated with Eq. (7)

(7) $$F=\frac{Between\;group\;variability}{within\;group\;variability}$$

$$\text{Null}\;\text{Hypothesis}=\left\{ \begin{array}{c} Rejected,\;F_{\text{static}}<F_{\text{critical}\;\text{value}}\\ Accepted,\;Otherwise \end{array}\right.$$

## Data description

Table 1 shows data from ten SSR markers taken from the genomes of eleven monkeys. NCBI (https://www.ncbi.nlm.nih.gov) provides the genome dataset. The results suggest that SSR identification with pattern matching was quite beneficial in revealing variation in chosen genome libraries. These SSR markers may be used to compare and quantify genomic similarities.

**Table 1:** Ten SSRs were identified from genome sequences.

Data Set	Chromosome name	No.of patterns identified
A.Nancymaae	chrUn	1,10,966
C.C.Imitator	chrUn	1,08,223
Cercocebus_atys	chrUn	1,45,189
M.Leucophaeus	chrUn	1,15,378
P. Paniscus	chrUn	1,27,150
R.Bieti	chrUn	1,19,993
R.Roxellana	chrUn	1,10,616
S.Boliviensis	chrUn	1,34,976
T.Syrichta	chrUn	1,31,908
C.A.Palliatus	chrUn	1,07,098
M.Nemestrina	chrUn	1,57,578
		13,69,075

**Fig. 2:**
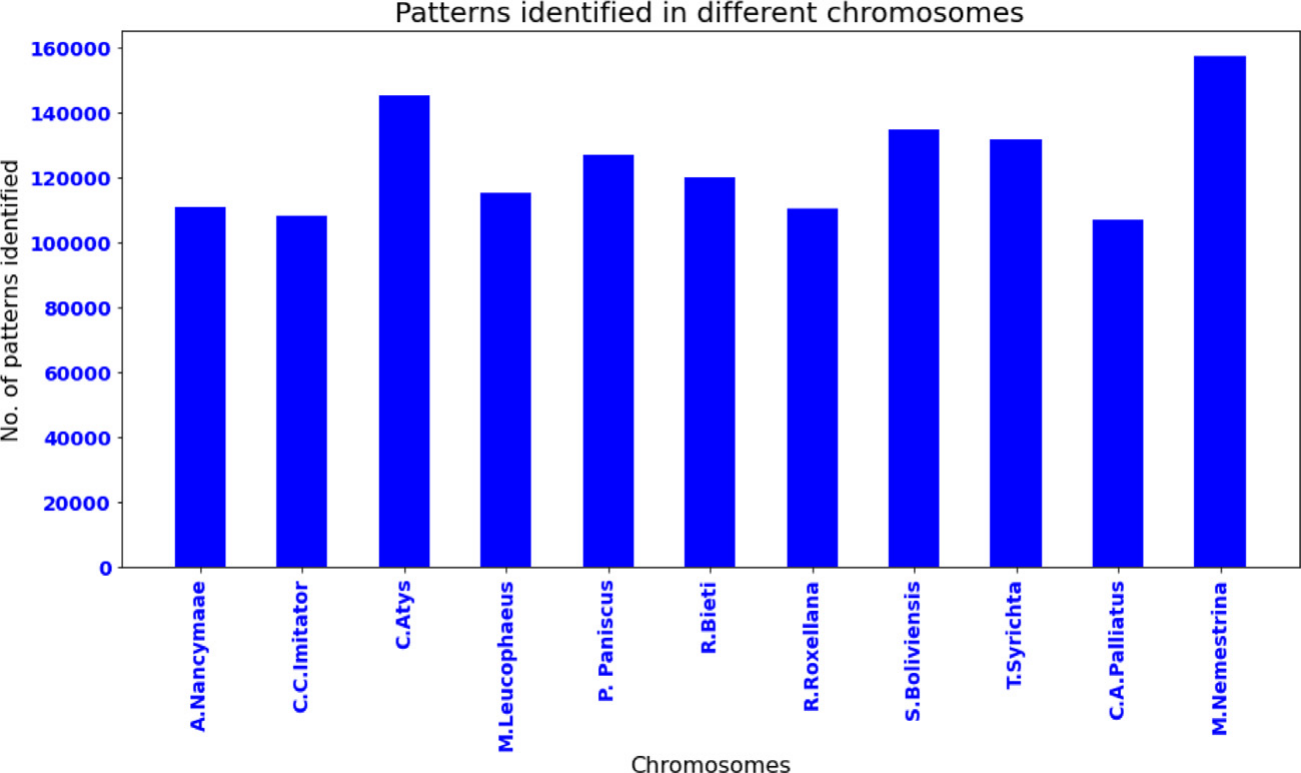
Patterns identified in different chromosomes.

Unchr chromosome of monkeys(*A.Nancymaae, C.C.Imitator, C.Atys, M.Leucophaeus, P. Paniscus, R.Bieti, R.Roxellana, S.Boliviensis, T.Syrichta, C.A.Palliatus and M.Nemestrina*) are taken into account for the identification of the 10 SSR markers listed in Table 1. The numbers of patterns identified in different chromosomes are depicted as the bar plot, which has been shown in Fig. 2.

Fig. A1 depicted in Appendix A (figures part) from A1(a) to (j)) has shown the max size of pattern related to considered genome datasets respectively. Fig. A2 depicted in Appendix A (figures part) from Fig. A2(a) to (k) has shown the Ten patterns related 11 datasets respectively.

## Statistical inference with ANOVA

### One-way analysis of variance

This method was employed to compare the averages of two or more samples (using the F distribution). This is only applicable to numerical response data (the "Y"), which is generally one variable, and numerical or (mostly) categorical input data (the "X"), which is always one variable, hence "One-way". One-way analysis of variance is performed among A.Nancymaae, C.C.Imitator, Cercocebus_atys, M.Leucophaeus, P. Paniscus,R.Bieti, R.Roxellana, S.Boliviensis, T.Syrichta, C.A.Palliatus and M.Nemestrina for Ten patterns. The actual results are shown in Tables 2 and 3.

**Table 2:** One-way ANOVA statistic and *p* value among 11 datasets.

Data Set	statistic	*P* value
A.Nancymaae	867.752255	0
C.C.Imitator	4411.909573	0
C.atys	19.92680516	9.56E-34
M.Leucophaeus	10783.28905	0
P. Paniscus	4237.052533	0
R.Bieti	4237.052533	0
R.Roxellana	13315.16044	0
S.Boliviensis	21076.54652	0
T.Syrichta	1671.161835	0
C.A.Palliatus	15423.71891	0
M.Nemestrina	11200.74466	0

**Table 3:** One-way ANOVA statistic and *p* value among 10 patterns among 11 datasets.

	statistic	*P* value
TAGA	815.3285535	0
AGAA	475.3548131	0
GATA	817.3908386	0
TCTA	423.9534899	0
TCAT	79.96858887	1.91E-148
GAAT	83.37284918	5.24E-155
AGAT	740.8585252	0
CTTT	149.8891403	4.32E-284
TATC	425.7145896	0
TCTG	66.91818823	5.45E-122

**Algorithm 1:** Complete_Heuristic Process.

**Complete_Heuristic (Text, Patt, ∑)**
1. n←Text.len#LengthofGenomeSequence
2. m←Patt.len#LengthofGenomepattern
3. α←First_Occurance_Position_Heuristic(Patt,m,∑)
4. β←Bad_Character_Heuristic(Patt,m,∑)
4. γ←Good_Suffix_Heuristic(Patt,m)
5. position←α
6. Whileposition≤n−−m
7. doj←m
8. Whilej>0andPatt[j]=Text[position+j]
9. doj←j−1
10. ifj=0then
11. *print ("Pattern occurs at shift", position)*
12. position←position+γ[0]
13. else
14. position←position+max(γ[j],j−β[Text[position+j]])

**Algorithm 2:** First Occurrence Position Heuristic Process.

**First_Occurance_Position_Heuristic (Patt, m, ∑)**
1. foreachpatternpatt∈patterns
α←0
2. fori←1tom
3. If[Patt[m−1]==Text[i+m−1]]
4. α←i
5. break;
6. else
7. continue;
5. Returnα

**Algorithm 3:** Bad Character Heuristic Process.

**Bad_Character_Heuristic (Patt, m, ∑)**
1. foreachcharactera∈∑
2. doβ[a]=0
3. fori←1tom
4. doβ[Patt[i]]←i
5. Returnβ

**Algorithm 4:** Good_Suffix_Heuristic Process.

**Good_Suffix_Heuristic (Patt, m)**
1. τ←identify_prefix(Patt)
2. $Patt^{\prime }\leftarrow \;reverse\;\left(Patt\right)$
3. $\tau^{\prime }\leftarrow$identify_prefix($Patt^{\prime }$)
4. forj←0tom
5. doγ[j]←m−τ[m]
6. fork←1tom
7. $do\;j\;\leftarrow \;m\;-\;\tau^{\prime }\left[k\right]$
8. $If\;\gamma \;\left[j\right]\;>\;l\;-\;\tau^{\prime }\left[k\right]\;then$
9. $\gamma \;\left[j\right]\;\leftarrow \;1\;-\;\tau^{\prime }\left[k\right]$
10. Returnγ

**Fig. 3:**
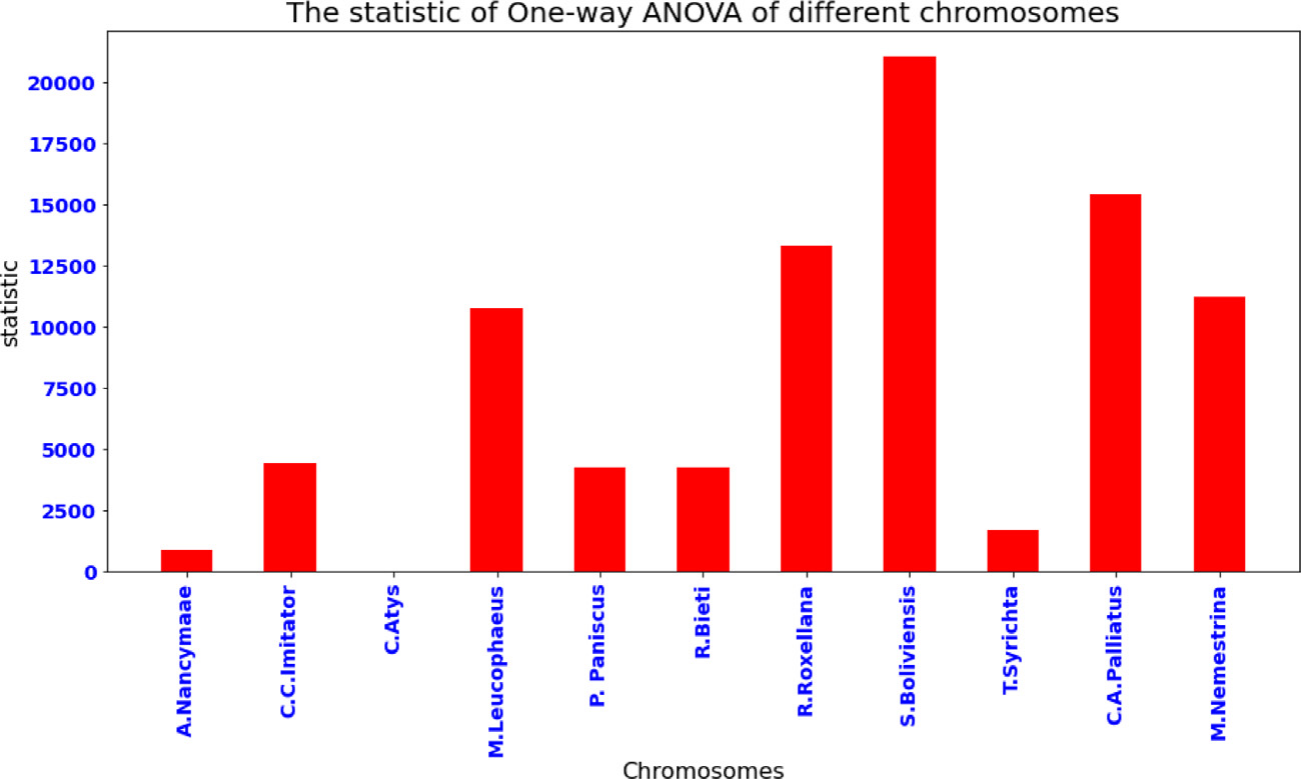
The statistic of One-way ANOVA of different chromosomes.

From Table 2, it is observed that null hypothesis is TRUE for every monkey dataset except C.Atys. So from these *p* value, we conclude that there is a similarity of C.Atys monkey with others. The statistic of One-way ANOVA of different chromosomes was depicted as the bar plot, which has been shown in Fig. 3.

**Fig. 4:**
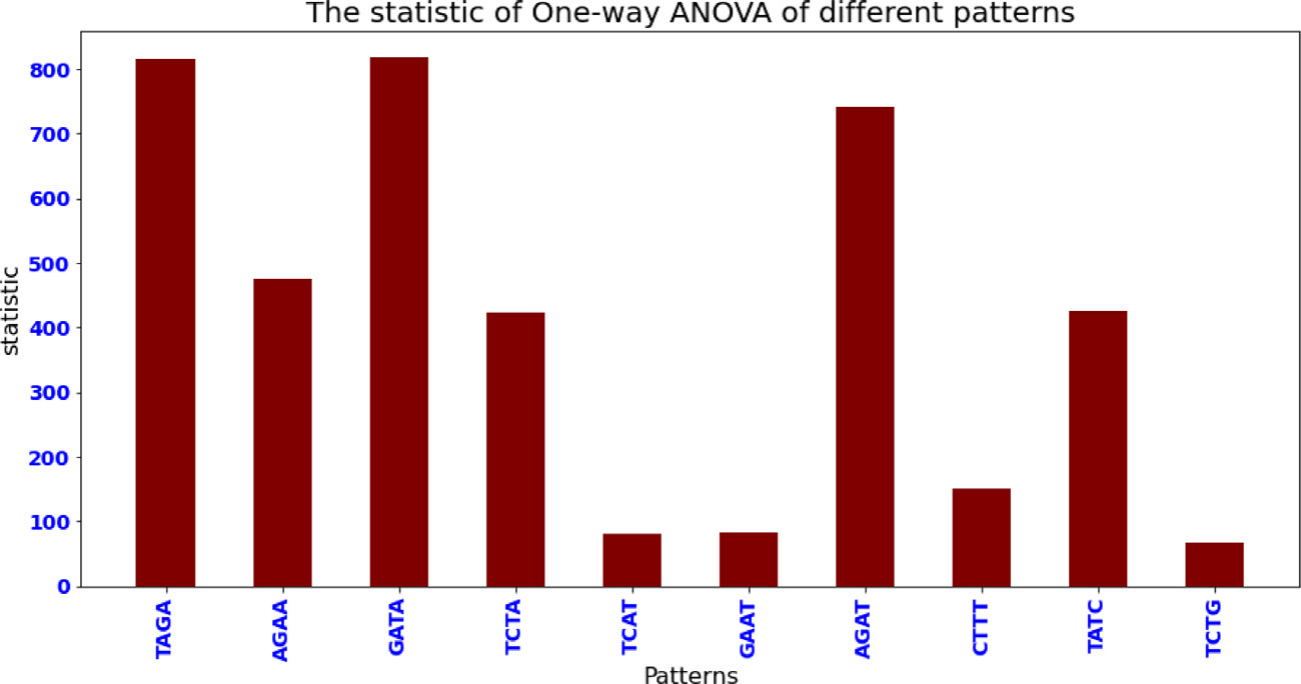
The statistic of One-way ANOVA of different patterns.

From Table 3, it is also observed that null hypothesis is TRUE for every pattern except four patterns called TCAT,GAAT,CTTT,TCTG. From these *p* value, we conclude that there is a similarity of TCAT,GAAT,CTTT,TCTG with others. The statistic of One-way ANOVA of different patterns was depicted as the bar plot, which has been shown in Fig. 4.

### Two-way analysis of variance

It looks at the impact of two categorical independent variables on a continuous dependent variable. It is used to determine not only the main impact of each independent variable, but also whether they interact. It is performed for each of 11 datasets (A.Nancymaae, C.C.Imitator, C.Atys, M.Leucophaeus, P.Paniscus, R.Bieti, R.Roxellana,S.Boliviensis, T.Syrichta, C.A.Palliatusand M.Nemestrina) among groups between the ten patterns.

The actual results are uploaded in mendeley Appendix A[source] & B[source].

Table A.1 to A.11 has shown the Two way ANOVA statistic and *p* value of 11 datasets for ten patterns. These results are shown the relation among monkey datasets interms of supporting the null hypothesis and other are alternate hypothesis. For example from the statistics and *p* value, it is observed that relation between TAGA and AGAA has alternative hypothesis, and TCTA and GAAT has null hypothesis.

Table A.12 to A.21 has shown the Two way ANOVA hypothesis reject TRUE/FALSE for 10 patterns of 11 datasets respectively. These results had shown that relation among patterns. For example relation AGAA b/w CTTT [meandiff: -0.1085, Lowet:0.135, Upper:0.3786] =>FALSE that means hypothesis reject False and for AGA b/w AGAT [meandiff-6.5503, Lowet: -6.7938, Upper: -6.3068] =>TRUE that means hypothesis reject True

Table B.1 to B.11 has shown the Two way ANOVA statistic and *p* value of individual ten patterns for 11 datasets. From table Table B.1 for TAGA pattern, it is observed that A.Nancymaae and C.C.Imitator has alternative hypothesis based its statistics and *p* value and A.Nancymaae and C.Atys has null hypothesis.

Table B.12 to B.21 has shown the two way ANOVA hypothesis reject TRUE/FALSE among 11 datasets related to ten patterns respectively. From table Table B.12 for TCAT pattern, it is observed that the relation between A.Nancymaae and C.C.Imitator [meandiff: 0.7039, Lowet: 0.4021, Upper: 1.0057]=>TRUE that means hypothesis reject True and for C.C.Imitator b/w S.Boliviensis[meandiff: -0.2713, Lowet: -0.573, Upper: 0.0305]=>FALSE that means hypothesis reject False.

**Fig. 5:**
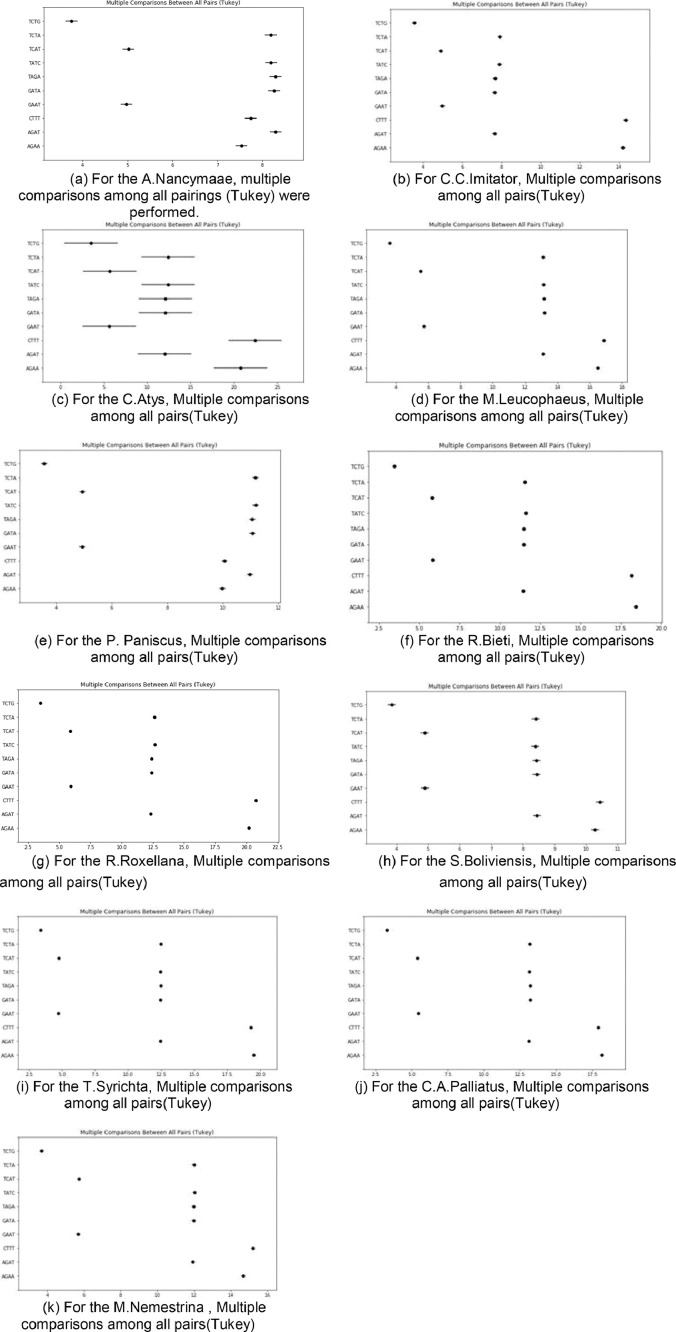
(a) For the A.Nancymaae, multiple comparisons among all pairings (Tukey) were performed. (b) For C.C.Imitator, Multiple comparisons among all pairs(Tukey). (c) For the C.Atys, Multiple comparisons among all pairs(Tukey). (d) For the M.Leucophaeus, Multiple comparisons among all pairs(Tukey). (e) For the P. Paniscus, Multiple comparisons among all pairs(Tukey). (f) For the R.Bieti, Multiple comparisons among all pairs(Tukey). (g) For the R.Roxellana, Multiple comparisons among all pairs(Tukey). (h) For the S.Boliviensis, Multiple comparisons among all pairs(Tukey). (i) For the T.Syrichta, Multiple comparisons among all pairs(Tukey). (j) For the C.A.Palliatus, Multiple comparisons among all pairs(Tukey). (k) For the M.Nemestrina, Multiple comparisons among all pairs(Tukey).

Fig. 5(a) to (k) has shown the Multiple comparisons between all pairs(Tukey) between 11 datasets for all 10 patterns. From the Fig. 5(a) to (k) it is observed that, 11 monkey dataset for 10 patterns, these graphs results matched with results discussed in the previous paragraphs.

**Fig. 6:**
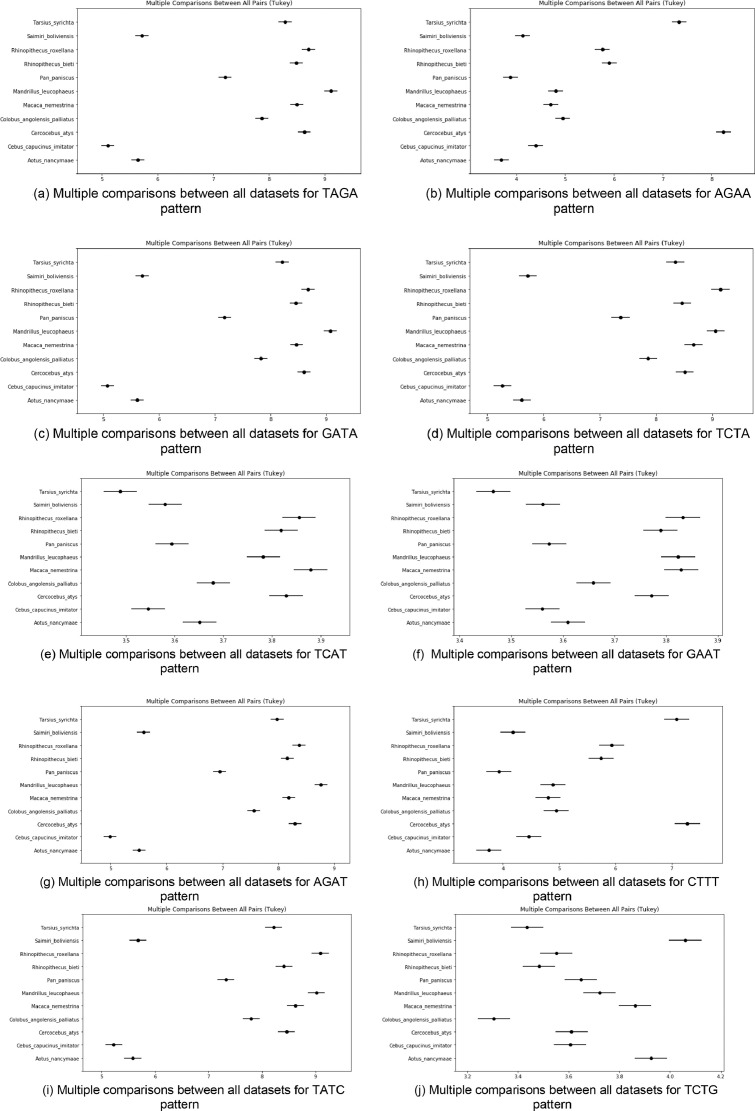
(a) Multiple comparisons between all datasets for TAGA pattern. (b) Multiple comparisons between all datasets for AGAA pattern. (c) Multiple comparisons between all datasets for GATA pattern. (d) Multiple comparisons between all datasets for TCTA pattern. (e) Multiple comparisons between all datasets for TCAT pattern. (f) Multiple comparisons between all datasets for GAAT pattern. (g) Multiple comparisons between all datasets for AGAT pattern. (h) Multiple comparisons between all datasets for CTTT pattern. (i) Multiple comparisons between all datasets for TATC pattern. (j) Multiple comparisons between all datasets for TCTG pattern.

**Fig. A1:**
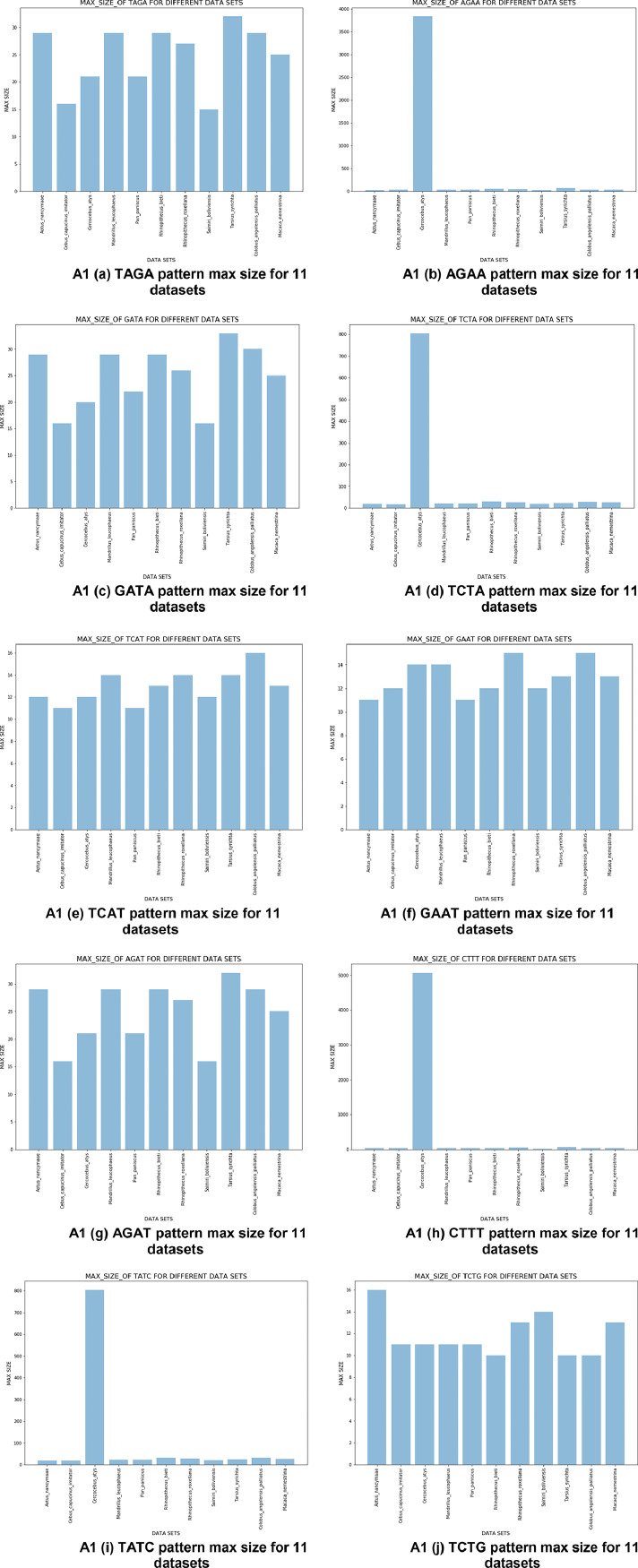
(a) TAGA pattern max size for 11 datasets. (b) AGAA pattern max size for 11 datasets. (c) GATA pattern max size for 11 datasets. (d) TCTA pattern max size for 11 datasets. (e) TCAT pattern max size for 11 datasets. (f) GAAT pattern max size for 11 datasets. (g) AGAT pattern max size for 11 datasets. (h) CTTT pattern max size for 11 datasets. (i) TATC pattern max size for 11 datasets. (j) TCTG pattern max size for 11 datasets.

**Fig. A2:**
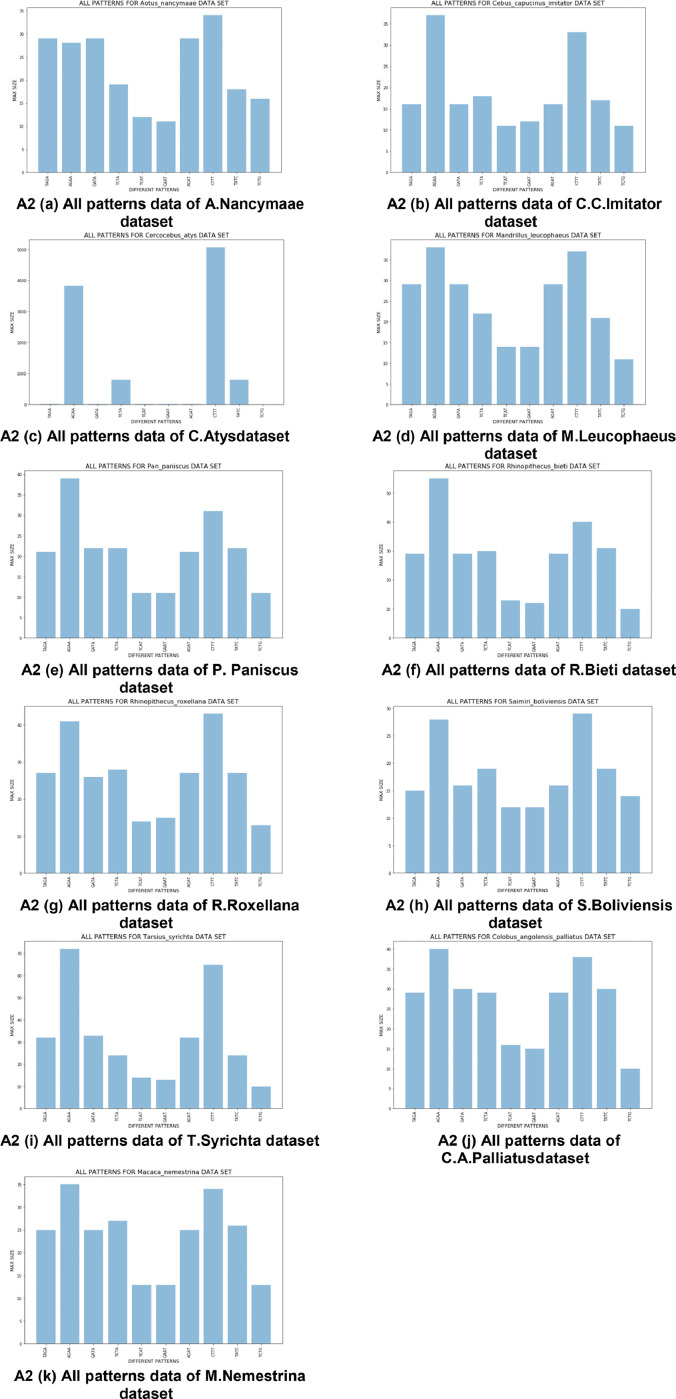
(a) All patterns data of A.Nancymaae dataset. (b) All patterns data of C.C.Imitator dataset. (c) All patterns data of C.Atysdataset. (d) All patterns data of M.Leucophaeus dataset. (e) All patterns data of P. Paniscus dataset. (f) All patterns data of R.Bieti dataset. (g) All patterns data of R.Roxellana dataset. (h) All patterns data of S.Boliviensis dataset. (i) All patterns data of T.Syrichta dataset. (j) All patterns data of C.A.Palliatusdataset. (k) All patterns data of M.Nemestrina dataset.

Fig. 6(a) to (j) has shown the Multiple comparisons between all pairs(Tukey) between 10 patterns for all 11 datasets. From the Fig. 6(a) to (j) it is also observed that, 10 patterns for 11 monkey dataset, these graphs results matched with results discussed in the previous paragraphs.

## Ethics statements

This work has never been published or submitted to another journal. This information and analysis will not hurt humans or animals.

## CRediT authorship contribution statement

**Chinta Someswara Rao:** Conceptualization, Methodology, Software, Writing – review & editing. **G.N.V.G. Sirisha:** Data curation, Writing – original draft. **K. Butchi Raju:** Visualization, Investigation. **N V Ganapathi Raju:** Supervision, Validation.

## Declaration of Competing Interests

The authors declare that they have no known competing financial interests or personal relationships that could have appeared to influence the work reported in this paper.

## Data Availability

Data will be made available on request.

## Appendix A

See Appendix Fig. A1, Fig. A2 here.
